# Maelstrom promotes tumor metastasis through regulation of FGFR4 and epithelial-mesenchymal transition in epithelial ovarian cancer

**DOI:** 10.1186/s13048-022-00992-4

**Published:** 2022-05-06

**Authors:** Wei-Peng He, Gui-Ping Yang, Zun-Xian Yang, Hong-Wei Shen, Ze-Shan You, Guo-Fen Yang

**Affiliations:** Department of Gynecology, the First Affiliated Hospital, Sun Yat-Sen University, No. 58, Zhongshan Road II, Guangzhou, 510080 China

**Keywords:** Ovarian cancer, Maelstrom, EMT, Metastasis, FGFR4

## Abstract

**Background:**

Increasing evidence has indicated that Maelstrom (MAEL) plays an oncogenic role in various human carcinomas. However, the exact function and mechanisms by which MAEL acts in epithelial ovarian cancer (EOC) remain unclear.

**Results:**

This study demonstrated that MAEL was frequently overexpressed in EOC tissues and cell lines. Overexpression of MAEL was positively correlated with the histological grade of tumors, FIGO stage, and pT/pN/pM status (*p* < 0.05), and it also acted as an independent predictor of poor patient survival (*p* < 0.001). Ectopic overexpression of MAEL substantially promoted invasiveness/metastasis and induced epithelial-mesenchymal transition (EMT), whereas silencing MAEL by short hairpin RNA effectively inhibited its oncogenic function and attenuated EMT. Further study demonstrated that fibroblast growth factor receptor 4 (FGFR4) was a critical downstream target of MAEL in EOC, and the expression levels of FGFR4 were significantly associated with MAEL. (*P* < 0.05).

**Conclusion:**

Our findings suggest that overexpression of MAEL plays a crucial oncogenic role in the development and progression of EOC through the upregulation of FGFR4 and subsequent induction of EMT, and also provide new insights on its potential as a therapeutic target for EOC.

## Introduction

Ovarian cancer is one of the most prevalent malignancies of the female genitalia and is the leading cause of cancer-related death among gynecological malignancies worldwide [1, 2]. Epithelial ovarian cancer (EOC) is the most common pathological subtype of ovarian cancers, accounting for approximately 90% of all ovarian cancers. Due to the lack of noticeable symptoms and early diagnosis strategies, patients with EOC are usually diagnosed at advanced stages (FIGO III or IV) and have an adverse prognosis. Although there have been advances in therapeutic options in recent decades, survival outcomes have yet to be substantively altered [3]. Metastasis to the pelvic and abdominal cavity is frequently observed in EOC patients and is considered a major factor in recurrent disease and unfavorable outcomes [4]. Despite the vast amount of clinical and basic research, specific reliable clinical/prognostic biomarkers are still quite limited and the mechanism of distal metastases has not been clarified. Hence, it is critical to identify genes involved in pelvic and abdominal metastasis and elucidate the related molecular mechanisms, with the ultimate goal of improving treatment efficacy and EOC patient outcomes.

The Maelstrom (MAEL) gene, located in 1q24, was first found in *Drosophila melanogaster* [5]. The encoded protein of MAEL is highly conserved, containing MAEL domain and a high-mobility group (HMG) domain, and this protein plays crucial roles in transcriptional transposon silencing mediated by piRNA in various species. The MAEL domain has an RNase H-like folding lacking a conserved classical catalytic residue like the one in RNase H-like superfamily nucleases, and it has an endonuclease activity specific for single stranded RNA (ssRNA) [6–9]. MAEL is only specifically expressed in normal testicular tissue, but is either not expressed or limitedly expressed in other normal tissues. In addition, it has been reported that MAEL is highly expressed in a variety of cancer cell lines, including lung cancer, liver cancer, breast cancer, bladder cancer, and colorectal cancer [9–12]. Furthermore, the high expression of MAEL has been correlated closely with epithelial-mesenchymal transition (EMT), tumor aggressiveness and poor patient prognosis [10–12]. However, the molecular mechanisms for aberrant MAEL expression in EOC remain unclear.

Herein, we sought to determine the roles of MAEL in tumorigenicity of EOC. We detected MAEL expression in both normal and EOC tissues, and evaluated the roles of MAEL as a clinical biomarker for EOC patients. To investigate the mechanisms underlying the potential oncogenic role of MAEL, we analyzed the effect of MEAL on EOC cell migration, invasion and proliferation, and examined its correlation with EMT in vitro and in vivo. Our results demonstrated the functional roles and underlying mechanisms of MAEL in the growth and metastasis of EOC.

## Results

### MAEL expression in EOC cell lines and human EOC tissues

**Fig. 1:**
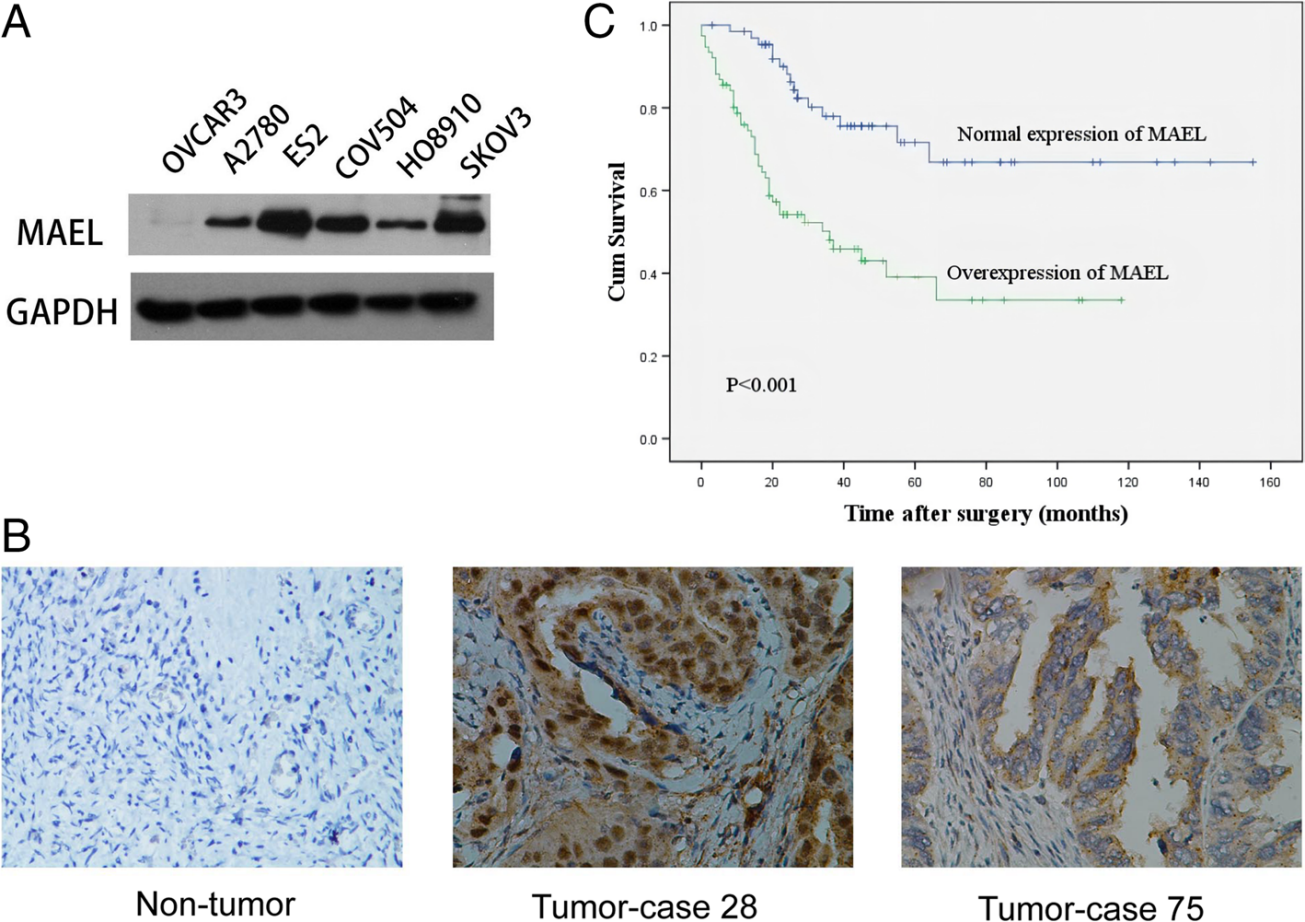
Expression of MAEL and its prognostic significance in EOC. **A** The MAEL protein expression was tested by Western blot in EOC cell lines. **B** Representative IHC staining images. Normal MAEL expression on surface epithelium of ovary was observed, and overexpression of MAEL in ovarian carcinoma was examined (case 28 and case 75) (400×). **C** Patients with overexpression of MAEL had a shorter survival period as determined by Kaplan-Meier survival analysis. Log-rank test was used to determine the statistical significance

To evaluate the expression of MAEL in EOC, western blot was used to analyze the MAEL levels in six EOC cell lines. Endogenous MAEL was overexpressed in ES2 and SKOV3 cell lines, whereas there were low levels in the other four EOC cell lines tested, HO8910, COV504, A2780, and OVCAR3, especially so in HO8910 and OVCAR3 cells (Fig. 1A). We further examined and compared MAEL levels in EOC, borderline tumors, benign cystadenomas and normal ovarian tissues using IHC analysis. The results revealed overexpression of MAEL in 53% (76/143) of EOC specimens, 30% (6/20) of borderline tumor specimens, and 10% (3/30) of cystadenoma specimens, but no overexpression in normal ovarian tissues (*p* < 0.001, Table 1, Fig. 1B).

**Table 1:** The expression of MAEL in normal ovaries and benign and malignant epithelial ovarian tumors^a^

		MAEL protein	
	All cases	Normal expression	Overexpression
**Normal ovaries**	30	30 (100%)	0 (0)
**Cystadenomas**	30	27 (90%)	3 (10%)
**Borderline tumors**	20	14 (70%)	6 (30%)
**Epithelial ovarian cancers**	143	67 (47%)	76 (53%)

^a^Values are n (%). A significantly increased frequency of intensive expression of MAEL was observed in borderline tumors, invasive carcinomas and cystadenomas (*P* < 0.001, Chi-Square Test for Trend)

### Overexpression of MAEL is linked to clinicopathological characteristics and predicts poor prognosis in EOC patients

The relationship between MAEL expression and several known clinicopathological features was subsequently investigated. Correlation analysis showed that overexpression of MAEL was positively correlated with the histological grade of tumors, FIGO stage, and pT/pN/pM status (*p* < 0.05, Table 2). No statistically significant correlations were observed between MAEL level and other clinicopathological features such as tumor histological type or patient age. Kaplan-Meier survival analysis showed that patients with high levels of MAEL exhibited a remarkably worse outcome (mean survival 54.5 months) than those with low levels of MAEL (mean survival 114.8 months) (*p* < 0.001, Fig. 1C). In addition, multivariate Cox regression analysis showed that MAEL upregulation was linked to poor survival of EOC patients, and therefore was an potential prognostic biomarker for overall survival of EOC patients (*p* < 0.01, Table 3).

**Table 2:** Correlation of MAEL expression with patients’ clinico-pathological characteristics in 143 ovarian carcinomas

		MAEL protein		
	All cases	Normal expression	Overexpression	***P*** value ^a^
Age at surgery (years)				0.547
≤ 51.5 ^b^	73	36 (49%)	37 (51%)	
> 51.5	70	31 (44%)	39 (56%)	
Histological type				0.696
Serous	97	45 (46%)	52 (54%)	
Mucinous	16	9 (56%)	7 (44%)	
Others^c^	30	13 (43%)	17 (57%)	
Histological grade (Silveberg)				< 0.001
G1	22	15 (68%)	7 (32%)	
G2	83	44 (53%)	39 (47%)	
G3	38	8 (21%)	30 (79%)	
pT status				0.032
pT1	41	25 (61%)	16 (39%)	
pT2	26	14 (54%)	12 (46%)	
pT3	76	28 (37%)	48 (63%)	
pN status				< 0.001
pN0	70	45 (64%)	25 (36%)	
pN1	73	22 (30%)	51 (70%)	
pM status				0.045
pMX	121	61 (50%)	60 (50%)	
pM1	22	6 (27%)	16 (73%)	
FIGO stage				0.004
I	25	18 (72%)	7 (28%)	
II	15	10 (67%)	5 (33%)	
III	81	33 (41%)	48 (59%)	
IV	22	6 (27%)	16 (73%)	

^a^Chi-square test

^b^Mean age

^c^Clear cell, Endometrioid, and Undifferentiated types

**Table 3:** Multivariate analysis of overall survival (Cox regression model)

Variable	Relative risk	95% Confidence interval	***P*** value
MAEL^a^	2.495	1.370–4.543	0.003
FIGO stage^b^	3.353	2.161–5.205	< 0.001

^a^Overexpressin vs Normal expression

^b^Stage IV vs Stage III vs Stage II vs Stage I

### Altered expression levels of MAEL impact migration and invasion of human EOC cells in vitro

**Fig. 2:**
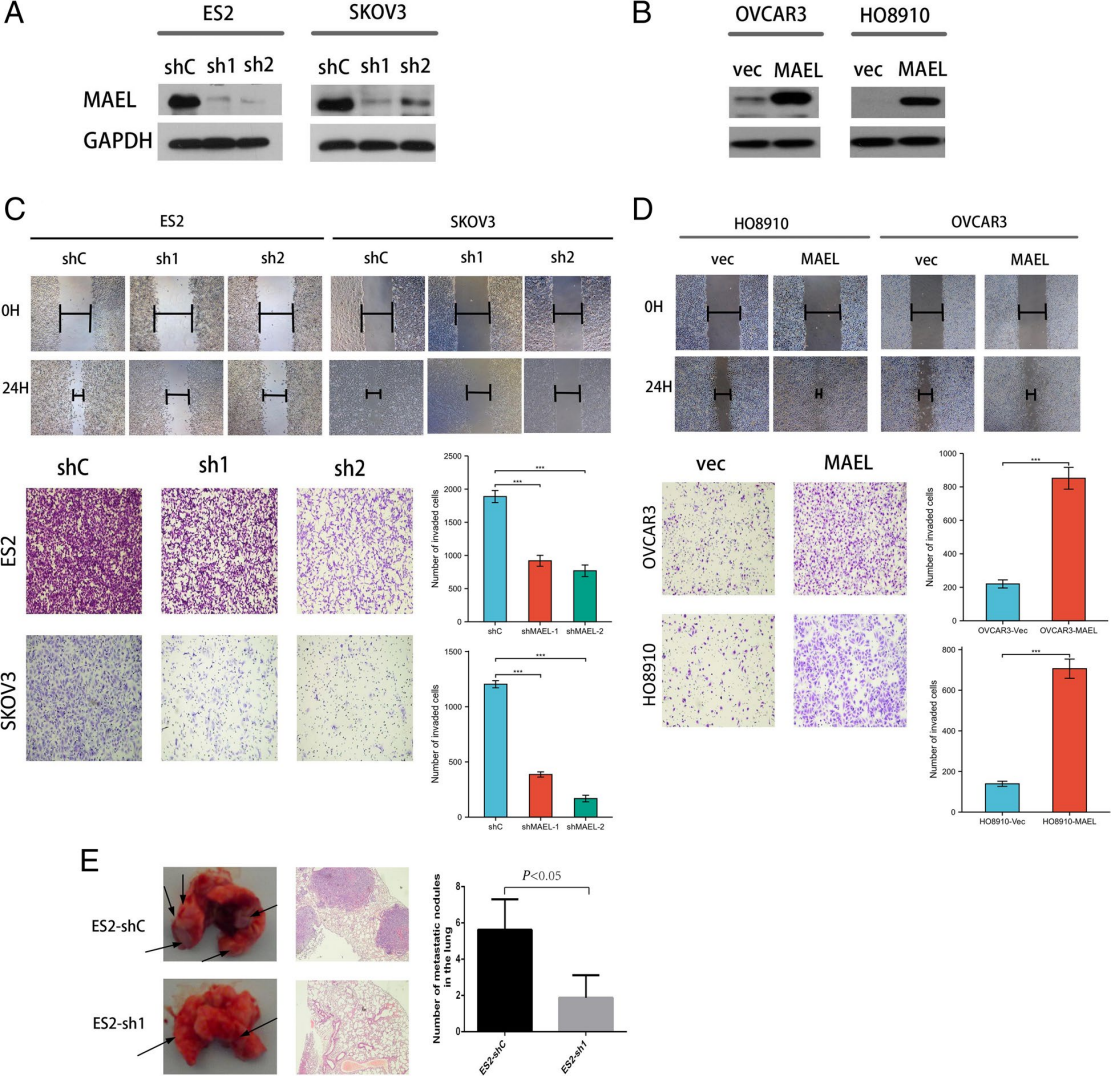
MAEL influences the invasion and metastasis of human EOC cells. **A** Western blot analysis confirmed that MAEL was effectively knocked down by transfection of MAEL-shRNAs into ES2 and SKOV3 cells. **B** Western blot analysis confirmed that MAEL was substantially increased in stable MAEL-transfected OVCAR3 and HO8910 cells.**C** Wound-healing showing the migration and transwell invasion assay showing the invasion of MAEL-shRNAs-transfected ES2 and SKOV3 cells by comparison with the corresponding control cells. Data are presented as the mean ± SD of three independent experiments (***p < 0.001). **D** Wound-healing showing the migration and transwell invasion assay showing the invasion of MAEL-transfected OVCAR3 and HO8910 cells by comparison with the corresponding control cells. Data are presented as the mean ± SD of three independent experiments (***p < 0.001). **E** Representative lungs with metastatic nodules (tagged by arrow heads) and lung metastatic tumors stained with hematoxylin and eosin (H&E). Number of lung metastatic nodules four weeks after injection of ES2-shMAEL-1 and ES2-shControl stable clones (*n* = 8, *p* < 0.05)

To investigate the oncogenic function of MAEL, ES2 and SKOV3 cells were stably transfected with two specific shRNAs targeting MAEL (shMAEL-1 and shMAEL-2) or a negative control shRNA (Fig. 2A). In addition, MAEL was also stably transfected into OVCAR3 and HO8910 cells (Fig. 2B). Subsequently, the stable MAEL-shRNAs-transfected and MAEL-transfected EOC cells were used for migration and invasion assays. Wound healing migration and transwell invasion assays showed that MAEL knockdown by shRNA significantly inhibited the migratory and invasive abilities of ES2 and SKOV3 cells (Fig. 2C). In contrast, ectogenic overexpression of MAEL dramatically enhanced cell migratory and invasive capacity in OVCAR3 and HO8910 cells as compared to the vector control cells (Fig. 2D).

### Silencing MAEL inhibits the metastasis of EOC in vivo

Next, we investigated whether MAEL influences the metastasis of ovarian carcinoma in vivo using a mouse xenograft model. We delivered ES2-shMAEL-1 and ES2-shControl stable clones into nude mice by tail vein injection. Upon 4 weeks post-injection, the numbers of metastatic nodules formed on the surface of lungs were counted after harvest. We found that silencing MAEL significantly inhibited the metastasis of EOC to the lung (Fig. 2E).

### MAEL induces EMT in EOC cells

**Fig. 3:**
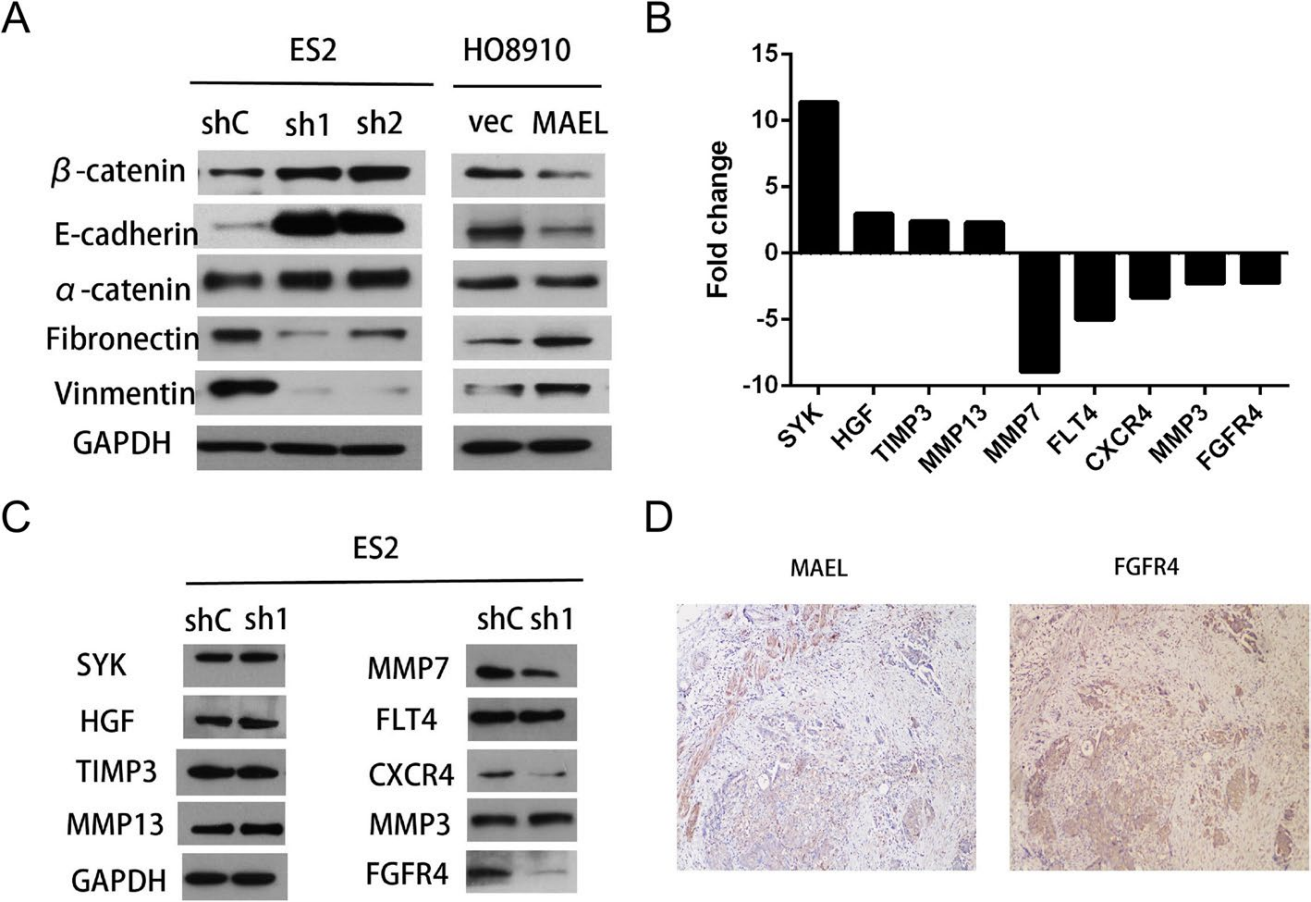
MAEL induces EMT and up-regulates FGFR4 expression. Western blot analysis showing that the expression of α-catenin, β-catenin and E-cadherin increased while the expression of vimentin and fibronectin decreased in MAEL-silenced ES2 cells, compared to those in corresponding control cells. The expression of α-catenin, β-catenin and E-cadherin decreased while the expression of vimentin and fibronectin increased in stable MAEL-transfected HO8910 cells compared to those in corresponding control cells. **B** Nine genes (i.e. SYK, HGF, TIMP3, MMP13, MMP7, FLT4, CXCR4, MMP3 and FGFR4) were found to exhibit a more than 2-fold different expression in ES2-shMAEL-1 cells compared to that in ES2-shControl cells using a Human Tumor Metastasis RT2 Profiler™ PCR Array. **C** The expression of SYK, HGF, TIMP3, MMP13, MMP7, FLT4, CXCR4, MMP3 and FGFR4 was verified in ES2-shMAEL-1 cells and their respective controls by western blot. **D** Overexpression of MAEL and FGFR4 were detected by IHC in a EOC case (200×)

Since EMT is a critical process in tumor metastasis, we subsequently investigated the expression of EMT markers in order to assess the influence of MAEL on EMT. Western blot analysis further affirmed that in the MAEL-silenced ES2 cells, the expression of mesenchymal markers (i.e. vimentin and fibronectin) was decreased while the expression of epithelial markers (i.e. α-catenin, β-catenin and E-cadherin) was increased (Fig. 3A). In contrast, there was a reduction in the expression of α-catenin, β-catenin and E-cadherin, whereas the expression of fibronectin and vimentin was elevated in HO8910 cells with ectogenic overexpression of MAEL (Fig. 3A).

### MAEL regulates FGFR4 expression in EOC cells

To investigate the downstream target genes regulated by MAEL and the mechanism underlying its promotion of EOC cell invasion and EOC metastasis, we examined the mRNA expression profiles in ES2-shControl and ES2-shMAEL-1 cells using a Human Tumor Metastasis RT2 Profiler™ PCR Array (Super Array Bioscience, America) containing 84 cell metastasis-related genes. Totally 9 genes exhibited a more than 2-fold higher expression in ES2-shMAEL-1 cells than in ES2-shControl cells. These included 4 upregulated genes, SYK, HGF, TIMP3, and MMP13 as well as 5 downregulated genes, MMP7, FLT4, CXCR4, MMP3, and FGFR4 (Fig. 3B). Consistent with these results, a western blot assay validated the protein expression levels of MMP7, CXCR4, and FGFR4 in ES2-shMAEL-1 cells (Fig. 3C).

Additionally, to validate the relevance of our cell line–based findings, we analyzed the correlations between the protein expression of MAEL and that of some of the downregulated genes, specifically MMP7, CXCR4 and FGFR4, using IHC. Results indicated that there was an evident association between the expression levels of MAEL and FGFR4 (*p* < 0.05, Table 4, Fig. 3D), however, no obvious association between MAEL and MMP7 or CXCR4 was observed.

**Table 4:** Association of MAEL expression with MMP7, CXCR4 and FGFR4 in 143 ovarian carcinomas

Variable		MAEL protein		
	All cases	Normal expression	Overexpression	***P*** value ^**a**^
**MMP7**				0.5033
Low expression	79	39(49%)	40 (51%)	
High expression	64	28 (44%)	36 (56%)	
**CXCR4**				0.2466
Low expression	82	35 (43%)	47 (57%)	
High expression	61	32 (52%)	29 (48%)	
**FGFR4**				0.0344
Low expression	63	38 (60%)	25 (40%)	
High expression	80	34 (42%)	46 (58%)	

^a^Chi-square test

## Discussion

Ovarian cancer has unique yet unfortunate characteristics of difficult early diagnosis and early metastasis [13, 14]. Therefore, identifying the molecular mechanisms of ovarian cancer metastasis is critical for the development of new targets and thus improving the outcome for EOC patients. Recently, it has been suggested that MAEL is an oncogene and a potential therapeutic target for a variety of cancers [10–12, 15]. However, the abnormal expression of MAEL and its functions in EOC have yet to be illuminated. This study demonstrated the frequent overexpression of MAEL in a series of EOC cell lines and tissues. Using IHC, we demonstrated that the overexpression of MAEL in EOC was closely related to pT/pN/pM status, tumor histological grade, and advanced FIGO stage, and also acted as a predictor of poor patient survival. These observations emphasize the critical roles of MAEL in aggressiveness and development of EOC.

The biofunctions of MAEL in EOC remain unclear thus far. This study explored the biological functions of MAEL in regulating EOC malignant phenotype. Results showed that MAEL silencing in EOC cells significantly attenuated while MAEL overexpression dramatically enhanced migration and invasion of EOC cells, respectively. Further in vivo experiment showed that enforced knockdown of MAEL resulted in inhibition of EOC metastasis in mouse model. In summary, these results demonstrated that MAEL is involved in EOC cell invasion and EOC metastasis, and has strong tumor-promoting effects in EOC. Our results were in agreement with previous studies, which showed that MAEL upregulation promotes tumor metastasis [10–12, 15], but opposite results were obtained by Lim et al., which showed that MAEL plays a tumor-suppressive role [16].

EMT is a crucial step in tumorigenesis and metastasis [17, 18]. Previous studies demonstrated that EMT plays a critical role in migration of ovarian cancer cells and metastasize of ovarian tumors [19–21]. Our study showed that overexpression of MAEL was significantly correlated with EOC cell migration and invasion. To further understand the oncogenic role of MAEL in EOC, we next explored the roles of MAEL in the EMT process. Expectedly, we found that MAEL could induce EMT, in which the ability of EMT was attenuated in ES2 cells with stably enforced knock down of MAEL. On the contrary, the reverse result was observed in O8910 cells with stably ectogenic overexpression of MAEL. These results shed new light on the mechanisms of MAEL-induced EMT in EOC metastasis.

As a new candidate oncogene, MAEL’s precise molecular mechanism in promoting cancer progression has yet to be clarified. A previous study has shown that MAEL can activate the AKT/GSK3beta/snail pathway to promote EMT of hepatocellular carcinoma cells and eventually cancer metastasis [10]. In bladder cancer, MAEL plays a critical oncogenic role in supporting cell EMT and invasion and bladder cancer metastasis via downregulation of MTSS1 through DNMT3B [11]. A study in colon cancer also suggests that MAEL can induce EMT and stemness characteristics to promote the invasion and metastasis of colon cancer [12]. To further explore the downstream genes regulated by MAEL involved in regulation of invasiveness and metastasis of EOC, we screened the differential gene expression profiles between ES2-shControl and ES2-shCHD1L-1 cells through a Human Tumor Metastasis real-time PCR array containing 84 cell invasion/ tumor metastasis-related genes. We discovered and identified 9 target genes which exhibited a more than 2-fold different expression. Consistently, we observed that MMP7, CXCR4, and FGFR4 were downregulated in ES2-shMAEL-1 cells as examined by western blot assay. Interestingly, we only observed a positive relationship between expression of MAEL and FGFR4 in our study. Taken together, these results propose that MAEL may promote EOC cell invasion and tumor metastasis via regulation of FGFR4.

FGFR4 is a fibroblast growth factor receptor with conserved amino acid sequence. It consists of an extracellular variant region, a single transmembrane region, a conservative region binding to heparan sulfate proteoglycan, an intracellular tyrosine kinase region, and an FGF-binding region. The protein’s extracellular domain partially binds to FGF, leading to conformational changes and activation of the STAT3 signaling pathway via autophosphorylation of its intracellular kinase domain. The autophosphorylation of FGFR4 can also phosphorylate its adapter protein FRS2α, then activate the Grb2/Sos1 complex thereby initiating downstream MAPK and PI3K/AKT signaling pathways [22]. However, FGFR4 exhibits variable oncogenic roles in different tumors. Prior reports showed that FGFR4 is highly expressed in many different cancers, such as lung cancer, colorectal cancer, liver cancer, esophageal cancer, gastric cancer, and ovarian cancer. FGFR4 can promote tumor cell invasion, proliferation and development of resistance, while FGFR4 inhibitors or silencing using siRNA can attenuate or reverse these effects. In addition, FGFR4 overexpression is closely correlated with poor survival of patients [23–30]. However, in other cancers the observations have been inconsistent [31–33]. Taking all of the above into consideration together with our findings on the functions of MAEL in EOC, it is proposed that FGFR4 might be a downstream target correlated with the aggressive behavior of MAEL-mediated EOC, and thus can promote cancer cell invasion and metastasis. Until now, the molecular mechanisms by which MAEL modulates the expression of FGFR4 remain unclear. Hence, it is obvious that further studies are required to elucidate how MAEL regulates the expression of FGFR4 and therefore promotes the metastasis of ovarian cancer.

## Conclusions

MAEL is overexpressed in EOC, and the upregulation of MAEL promotes tumor cell migration, invasion, and metastasis by inducing EMT. Moreover, we present evidence that MAEL promotes tumor invasion and metastasis through upregulation of FGFR4. These findings highlight the critical function of MAEL in the tumorigenesis of EOC and the resulting aggressive/poor prognostic phenotype, and also provide new insights on its potential as a therapeutic target for EOC.

### Materials and methods

#### Patients and tissue microarray

Tissue samples were obtained from 193 epithelial ovarian tumors (carcinomatous, borderline, and benign). The samples were paraffin-embedded tissues archived between 1993 and 2004 at the Department of Pathology, the First Affiliated Hospital, Sun Yat-Sen University (Guangzhou, China) for construction of ovarian tissue microarray (TMA) as described previously [34]. Patients who died from emergency or unknown reasons or accepted preoperative anticancer treatment were excluded. All patients involved in this study provided informed consent and this study was approved by the Medical Ethics Committee of Sun Yat-Sen University.

#### Immunohistochemistry staining

The immunohistochemical (IHC) analyses were carried out as described previously [34]. TMA slides were first boiled in EDTA buffer (pH 8.0) to expose antigen, followed by incubation with antibodies against MAEL (1:100 dilution; Sigma, USA) or fibroblast growth factor receptor 4 (FGFR4) (1:200 dilution; Abcam, Cambridge, MA) at 4 °C overnight. The results of MAEL IHC staining were scored using a semiquantitative method that combined the staining intensity with the positive staining area. The staining index (divided into 0–12) was calculated according to staining intensity (strong = 3, moderate = 2, weak = 1, and negative = 0) and multiplied by the proportion of positively stained cells (< 25% = 1, ≥25 - < 50% = 2, ≥50 - < 75% = 3, and ≥ 75% = 4). The positive expression of MAEL was mainly in the cytoplasm of ovarian cells. The staining results were evaluated by two senior pathologists (Drs. J. Chen and D. Xie) in a double-blind manner.

#### Cell culture

The human ovarian cancer cell lines ES2, SKOV3, COV504, HO8910, A2780, and OVCAR3 were generously donated by the Department of Gynecology as a gift. ES2, HO8910, and SKOV3 cells were cultured in RPMI-1640 medium (GIBCO). The COV504, A2780, and OVCAR3 cells were cultured in Dulbecco’s Modified Eagle Medium (GIBCO). Both media were supplemented with 10% fetal bovine serum (FBS) (GIBCO), 100 U/mL penicillin and 100 μg/mL streptomycin and all the cells were cultured in a humidified incubator at 37 °C containing 5% CO_2_.

#### Western blot

Proteins extracted from cells using Radio-Immunoprecipitation Assay (RIPA) lysis buffer were quantified with Bicinchoninic Acid (BCA) assay (Thermo Fisher Scientific, Grand Island, NY, USA). Equal amounts of proteins were separated by sodium dodecyl sulfate-polyacrylamide gel electrophoresis (SDS-PAGE) and transferred onto polyvinylidene difluoride (PVDF) membranes (Millipore, Danvers, MA, USA), followed by incubation overnight at 4 °C with primary antibodies against the following human proteins: MAEL (Sigma, USA; 1:1000 dilution), α-catenin, β-catenin, Vimentin, E-cadherin, Fibronectin (all from BD Transduction Laboratories, Franklin Lakes, NJ; 1:1000 dilution), SYK, HGF, TIMP3, MMP13, MMP7, FLT4, CXCR4, MMP3, FGFR4 (all from Cell Signaling Technology; 1:1000 dilution), and GAPDH (Proteintech; 1:5000 dilution). The signals were visualized using an enhanced chemiluminescence kit (Amersham Biosciences, Uppsala, Sweden) and quantitated using Quantity One (Bio-Rad, Hercules, CA).

#### Plasmid construction and transfection

The full-length human MAEL cDNA was amplified and cloned into a pcDNA3.1 (+) expression vector (Invitrogen) to generate an overexpression vector as described previously [10]. Then, the expressed plasmids were transfected into OVCAR3 and HO8910 cells using Lipofectamine 2000 (Invitrogen) with an empty pcDNA3.1(+) vector as control. Stable MAEL-expressing clones were obtained by selection with Geneticin (Roche Diagnostics, Indianapolis, IN, USA), and the level of MAEL expression was confirmed by western blotting.

#### Construction of the recombinant lentiviral vector

To suppress MAEL expression, the lentivirus expression vector was constructed with the psi-LVRH1MP (GeneCopoeia Company, Rockville, MD, USA) shRNA expression system, according to the standard protocols. The targeting sequences of the lentiviral shRNA constructs are listed as follows: shMAEL-1: 5′-GGAACTGGCCACCTATCTACT-3′ and shMAEL-2: 5′-GAGTCAACTGGTGTTTGAAGC-3′. The HSH021057-LVRH1MP vector used as the shRNA control vector was also purchased from GeneCopoeia Company. The ES2 and SKOV3 cells were transfected with the constructed retroviruses. Stably infected cells were selected using puromycin (Santa Cruz Biotechnology, Santa Cruz, CA, USA), and the knockdown efficiency was quantified by western blotting.

#### In vitro cell migration and invasion assays

For wound-healing assay, the EOC cells were seeded in 6-well plates and cultured until reaching confluent. Then, wounds were generated using a 200 μl pipette tip. Cell migration into the wound area 24 h after scratching was photographed under an inverted phase-contrast microscope (Nikon, Tokyo, Japan) and measured. The transwell invasion assay was conducted using 24-well plates with Matrigel Invasion Chambers (BD Biosciences, Franklin Lakes, NJ). After a 24-h incubation, non-invading cells were removed, the migrated cells were fixed in methanol and stained with 10% Giemsa, followed by quantification under an inverted microscope. Experiments were repeated independently in triplicate.

#### Mouse xenograft assay

For the in vivo experiments, female BALB/c nude mice (4-weeks old) were purchased from Beijing Weitong Lihua Experimental Animal Technology Co. Ltd. (Beijing, China), randomly grouped and injected with 1 × 10^6^ ES2-sh1 and ES2-shC cells via the tail vein. Four weeks after injection, all the mice were sacrificed. The lungs were dissected and the metastatic nodules on the surface of the lungs were counted. All the animal experimental procedures were performed by following the NIH Guide for the Care and Use of Laboratory Animals (2011) and approved by the Animal Ethical and Welfare Committee (AEWC).

#### Real-time PCR gene array

The total RNA was extracted using Trizol (Invitrogen) and reverse-transcribed into cDNA using Super-Script III Reverse Transcriptase (Invitrogen). After cDNA was synthesized, amplification of cDNA was performed by PCR using the 2 × Super Array PCR master mix (SuperArray Bioscience, Frederick, MD). The Human Tumor Metastasis RT^2^ Profiler™ PCR Arrays (Super Array Bioscience) were analyzed using real-time PCR conducted on an Opticon™ DNA Engine ABI PRISM7900 system (Applied Biosystems, Foster City, CA). All data were normalized to GAPDH using the ΔΔCt method.

### Statistical analysis

All statistical analyses were carried out using the SPSS statistical software package (SPSS Standard version 20.0, SPSS Inc. Chicago, IL). For functional assay, all experiments were repeated three times. Data derived from cell and xenograft experiments are expressed as the mean ± standard deviation (SD) and were analyzed using two-tailed Student’s t-test. The Fisher’s exact test or chi-square test was used to assess differences between gene expression and clinicopathological features of the EOC patients. Kaplan–Meier method was used to plot the survival curves. The Cox proportional hazards model and Log-rank test were used to compare differences in survival between different patient groups. *P* < 0.05 was considered statistically significant.

## Data Availability

Not applicable.

## Abbreviations

MAEL: Maelstrom
FGFR4: Fibroblast growth factor receptor 4
EOC: Epithelial ovarian cancer
EMT: Epithelial-mesenchymal transition
TMA: Tissue microarray
IHC: Immunohistochemical
